# Organosiloxane-Modified Auricularia Polysaccharide (Si-AP): Improved High-Temperature Resistance and Lubrication Performance in WBDFs

**DOI:** 10.3390/molecules29112689

**Published:** 2024-06-06

**Authors:** Fan Zhang, Yu Wang, Bo Wang, Yuan Geng, Xiaofeng Chang, Wenzhe Zhang, Yutong Li, Wangyuan Zhang

**Affiliations:** 1 School of Petroleum Engineering, Xi’an Shiyou University, Xi’an 257015, China; 23211010046@stumail.xsyu.edu.cn (Y.W.); 23212010104@stumail.xsyu.edu.cn (Y.L.); 22111010005@stumail.xsyu.edu.cn (W.Z.); 2 Shaanxi Yanchang Petroleum (Group) Co., Ltd., Xi’an 710069, China; wangbo_ycp@163.com (B.W.); eagle.1983@163.com (W.Z.); 3 CNPC Engineering Technology R&D Co., Ltd., Beijing 102206, China; gengyuandr@cnpc.com.cn; 4 Chuanqing Drilling Engineering Company Ltd., Xi’an 710018, China; zjsyfcxf@cnpc.com.cn

**Keywords:** water-based drilling fluids, synthetic procedures and characterization, molecular diversity, high-temperature performance, organosiloxane-modified auricularia polysaccharide

## Abstract

This study introduces a novel organosilicon-modified polysaccharide (Si-AP) synthesized via grafting and comprehensively evaluates its performance in water-based drilling fluids (WBDFs). The molecular structure of Si-AP was characterized using Fourier-transform infrared spectroscopy (FTIR) and ^1^H-NMR experiments. Thermalgravimetric analysis (TGA) confirmed the good thermal stability of Si-AP up to 235 °C. Si-AP significantly improves the rheological properties and fluid loss performance of WBDFs. With increasing Si-AP concentration, system viscosity increases, API filtration rate decreases, clay expansion is inhibited, and drilling cuttings hydration dispersion is suppressed, especially under high-temperature conditions. Additionally, mechanistic analysis indicates that the introduction of siloxane groups can effectively inhibit the thermal degradation of AP chains and enhance their high-temperature resistance. Si-AP can form a lubricating film by adsorbing on the surface of clay particles, improving mud cake quality, reducing the friction coefficient, and significantly enhancing the lubricating performance of WBDFs. Overall, Si-AP exhibits a higher temperature-resistance limit compared to AP and more effectively optimizes the lubrication, inhibition, and control of the filtration rate of WBDFs under high-temperature conditions. While meeting the requirements of drilling fluid systems under high temperatures, Si-AP also addresses environmental concerns and holds promise as an efficient solution for the exploitation of deep-seated oil and gas resources.

## 1. Introduction

With the continuous growth in global energy demand, the exploration and development of oil and gas are advancing toward deeper and higher temperature regions, posing significant challenges to drilling operations [1,2,3]. The formulation and performance of drilling fluids are essential components in the drilling process, directly impacting the smooth progress of the entire drilling operation [4,5,6,7]. Polysaccharide compounds, such as cellulose, starch, Arabic gum, and guar gum, are widely used in drilling fluid systems due to their excellent thickening, dispersing, and emulsifying properties in aqueous solutions, thereby serving functions such as thickening, suspension, and lubrication. Among them, cellulose derivatives like hydroxyethyl cellulose (HEC) and hydroxypropyl methyl-cellulose (HPMC), as well as polysaccharide additives like guar gum, find extensive applications in drilling fluids due to their excellent thickening, dispersing, and lubricating properties [8,9,10,11,12]. However, as oil and gas exploration and development move toward deeper and higher-temperature regions, the performance of traditional plant polysaccharides at high temperatures has become inadequate [13,14,15,16,17,18]. On one hand, high temperatures can induce thermal degradation of polysaccharide molecules, leading to a significant decrease in key properties such as thickening and dispersing. On the other hand, the lubricity of polysaccharides at high temperatures also decreases significantly, increasing the friction between drill string and wellbore walls, thereby exacerbating the circulation resistance of the drilling fluid, affecting drilling efficiency, and potentially leading to safety hazards such as sticking and wear of the drill string and pipelines [1,4,11,19,20,21,22]. Therefore, the development of new additives that simultaneously enhance the heat resistance and environmental performance of polysaccharides has become one of the key issues urgently needing to be addressed in the field of drilling fluids.

In response to the above issues, researchers in recent years have turned their attention to organosilicon compounds [23,24,25,26,27]. Compared to other high-temperature stabilizers, organosilicon compounds, due to their unique molecular structure and excellent thermal stability, have shown broad prospects for applications in improving the heat resistance and lubricity of polysaccharides under high-temperature conditions [9,28,29,30]. Specifically, the introduction of organosilicon groups into Auricularia polysaccharide (Si-AP) can effectively inhibit the thermal degradation of polysaccharide chains, enhancing their thermal stability at high temperatures. The organosilicon groups can also regulate the hydrophilicity and hydrophobicity of Si-AP, endowing it with excellent lubricity and significantly improving the lubricating performance of water-based drilling fluids under high-temperature conditions. The lubricating film formed by Si-AP can reduce the contact area between the drill string and wellbore walls, thereby greatly reducing the friction coefficient and effectively preventing safety issues such as drill string sticking [31,32]. Furthermore, organosilicon compounds can form stable polysaccharide structures with polysaccharide molecules through hydrogen bonds, hydrophobic interactions, etc., further enhancing the thermal stability of polysaccharides and regulating their solubility and rheological properties in aqueous solutions to improve their thickening, suspension, and other performance under high-temperature conditions [27,33,34].

In our previous studies, we have established that Auricularia polysaccharides (AP) exhibit superior high-temperature performance and lubricity compared to other natural polysaccharides owing to their distinct molecular structure and properties. Leveraging this foundation, in the present work, we introduced organosilicon groups into AP via graft modification techniques to develop Si-AP, aiming to further enhance its thermal stability and lubricity under high-temperature conditions. We then comprehensively evaluated the performance of Si-AP in water-based drilling fluids (WBDFs) and investigated the underlying mechanism behind its improved high-temperature capabilities, which represents a novel and innovative approach compared to previous studies on polysaccharide-based high-temperature stabilizers.

## 2. Results and Discussion

### 2.1. Structural Characterizations of Si-AP

#### 2.1.1. FTIR Spectroscopy Analysis

**Figure 1:**
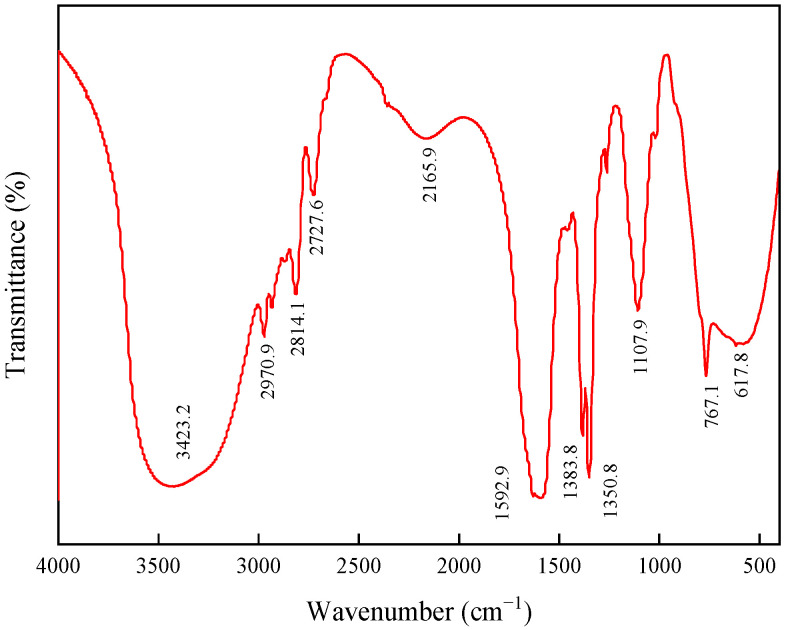
FTIR spectroscopy of Si-AP.

As shown in Figure 1, we conducted a structural analysis of Si-AP using a Fourier-transform infrared spectrometer:

The peak at 3423.2 cm^−1^ attributed to the stretching vibration of hydroxyl (–OH) groups on the polysaccharide chains of AP and the peak at 2970.9 cm^−1^ attributed to the stretching vibration absorption peak of Si–CH_3_ methyl groups, indicating the presence of organic silicon groups in Si-AP.

Additionally, the peak at 2814.1 cm^−1^ attributed to the stretching vibration of methyl (–CH_3_) or methylene (–CH_2_–) groups; the peak at 2727.6 cm^−1^ attributed to the asymmetric stretching vibration of methyl (–CH_3_) or methylene (–CH_2_–) groups, suggesting the presence of alkyl chain segments in Si-AP; and the peak at 2165.9 cm^−1^ attributed to the stretching vibration of silicon hydride (–Si–H).

Furthermore, the peak at 1592.9 cm^−1^ attributed to the stretching vibration absorption of C=C bonds, indicating the possible presence of aromatic structural units in the sample; peaks at 1383.8 cm^−1^ and 1350.8 cm^−1^ attributed to the deformation vibration of methyl (–CH_3_) or methylene (–CH_2_–) groups, indicating the presence of organic silicon groups in Si-AP; peaks at 1107.9 cm^−1^ and 767.1 cm^−1^ attributed to the stretching vibration of siloxane groups (Si–O–Si); and the peak at 617.8 cm^−1^ attributed to the stretching vibration of silicon–carbon bonds (–Si–C–), characteristic of organosilicon compounds.

These characteristic peaks confirm the molecular structural features of Si-AP samples, including the presence of organic silicon frameworks, aromatic rings, alkyl groups, and polysaccharide chains. The experimental results indicate a successful reaction between organic silicon monomers and AP, leading to the formation of a new composite material.

#### 2.1.2. ^1^H-NMR Analysis

**Figure 2:**
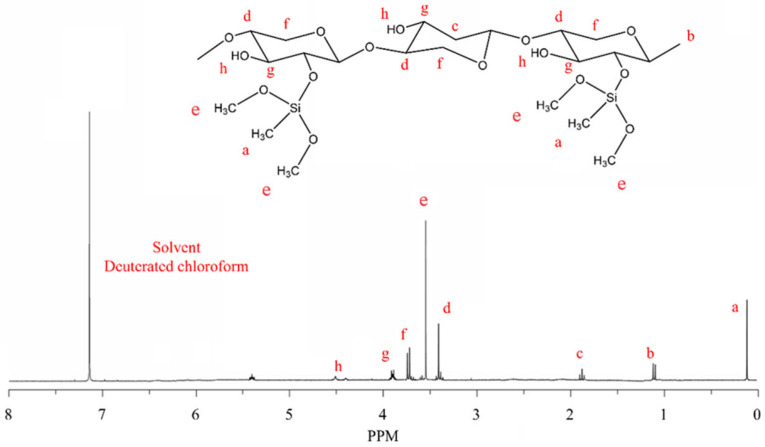
^1^H-NMR of Si-AP.

As shown in Figure 2, the characteristic peak at 0.12 ppm corresponds to the hydrogen atoms on the methyl group connected to the silicon atom in dimethoxydimethylsilane (DMVS), the characteristic peak at 1.11 ppm corresponds to the hydrogen atoms on the methyl (–CH_3_) group in the main chain of Si-AP, and the characteristic peak at 1.88 ppm corresponds to the hydrogen atoms on the methylene (–CH_2_–) group in the main chain of Si-AP.

The characteristic peak at 3.40 ppm corresponds to the hydrogen atoms on the secondary methyl (–CH–O–) group connected to the methylene group in Si-AP, the characteristic peak at 3.55 ppm corresponds to the hydrogen atoms on the methoxy (–OCH_3_) group in DMVS, and the characteristic peak at 3.73 ppm corresponds to the hydrogen atoms on the methylene oxide (–CH_2_–O–) group connected to the oxygen atom in Si-AP.

The characteristic peak at 3.90 ppm corresponds to the hydrogen atoms on the secondary methyl (–CH–OH) group connected to the hydroxyl (–OH) group in Si-AP, the characteristic peak at 4.51 ppm corresponds to the hydrogen atoms on the hydroxyl (–OH) group in Si-AP, and the characteristic peak at 5.40 ppm corresponds to the hydrogen atoms on the secondary methyl (–CH–O–) group connected to the oxygen atom in Si-AP. Additionally, the characteristic peak at 7.26 ppm corresponds to the deuterated solvent chloroform (CDCl_3_) used.

Analysis of the proton nuclear magnetic resonance peaks confirms the covalent bonding of dimethoxydimethylsilane to the main chain of AP, successfully preparing the desired Si-AP.

#### 2.1.3. Thermogravimetric Analysis

**Figure 3:**
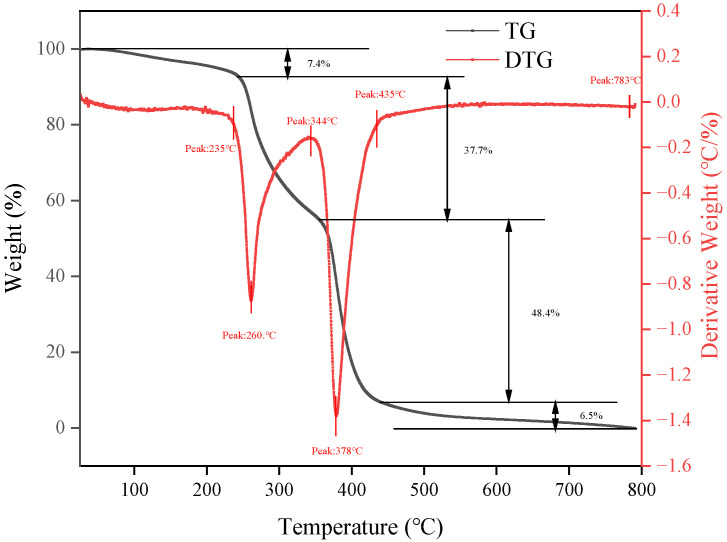
Thermogravimetric curve of Si-AP.

From the TG curve in Figure 3, it can be observed that before 235 °C, there is an initial weight loss attributed to moisture evaporation, with Si-AP exhibiting a weight loss of 7.4%. From 235 to 435 °C, the main thermal decomposition reaction of Si-AP occurs, with a weight loss of 86.1%. This is attributed to the thermal degradation of the polysaccharide structure and the organosilicon-modified structure in Si-AP. Above 435 °C, Si-AP essentially stabilizes into a residual state, with the remaining components comprising the more stable inorganic components of the Si-AP sample, such as the silicon framework. The DTG curve in Figure 3, representing the first derivative of the thermal weight change, more clearly illustrates the key temperature points of thermal decomposition. Si-AP exhibits two peak values at 260 °C and 378 °C, corresponding to the main thermal decomposition stages of the sample. Through the DTG curve, we can further determine the thermal stability of Si-AP and its potential range of thermal application temperatures. The thermal gravimetric experiment results indicate that Si-AP exhibits good thermal stability up to 235 °C.

#### 2.1.4. GPC Analysis

Utilizing gel permeation chromatography (GPC), we analyzed the molecular weight distribution characteristics of Si-AP. Experimental data (Table 1) revealed that the number-average molecular weight (Mn) of Si-AP was 40,966 g/mol, the weight-average molecular weight (Mw) was 106,619 g/mol, the z-average molecular weight (Mz) was 226,530 g/mol, and the polydispersity index (Mw/Mn) was 2.602604. These results indicate that Si-AP possesses a broad molecular weight distribution. The higher molecular weight characteristics are advantageous for enhancing the thickening and suspension stability of drilling fluid systems under demanding conditions such as high temperature and high shear. Additionally, the higher molecular weight implies that Si-AP consists of long polymer chains, thereby forming a stronger three-dimensional network structure, which better prevents the precipitation of drilling fluid particles.

### 2.2. Performance Evaluations

#### 2.2.1. Rheological Properties and Filtration Characteristics Tests

As shown in Table 2, with the increase in Si AP content, the apparent viscosity (AV), plastic viscosity (PV), and yield point (YP) of the base mud increase. For example, after adding 1.0% Si AP, the AV at room temperature increased by 5.7 times, PV increased by 8.2 times, and YP increased by 4 times. Meanwhile, these key rheological parameters also exhibit the same trend at high temperatures, reflecting the potential of Si AP to increase viscosity in drilling fluids under high-temperature conditions. This may help improve the suspension and transportation performance of base mud under high-temperature and high-shear conditions.

Furthermore, the incorporation of Si-AP into the water-based mud led to a substantial reduction in mud cake thickness. At 25 °C, the addition of 1.0% Si-AP decreased the mud cake thickness from 2.5 mm to 1.8 mm. At 180 °C, the addition of 1.0% Si-AP reduced the mud cake thickness from 2.8 mm to 2.0 mm. The reduction in mud cake thickness achieved through the incorporation of Si-AP indicates its ability to effectively inhibit the deposition and excessive formation of drill cuttings, thereby improving the filterability of the drilling fluid and minimizing potential borehole stability issues and flow restriction problems associated with excessive mud cake formation.

**Figure 4:**
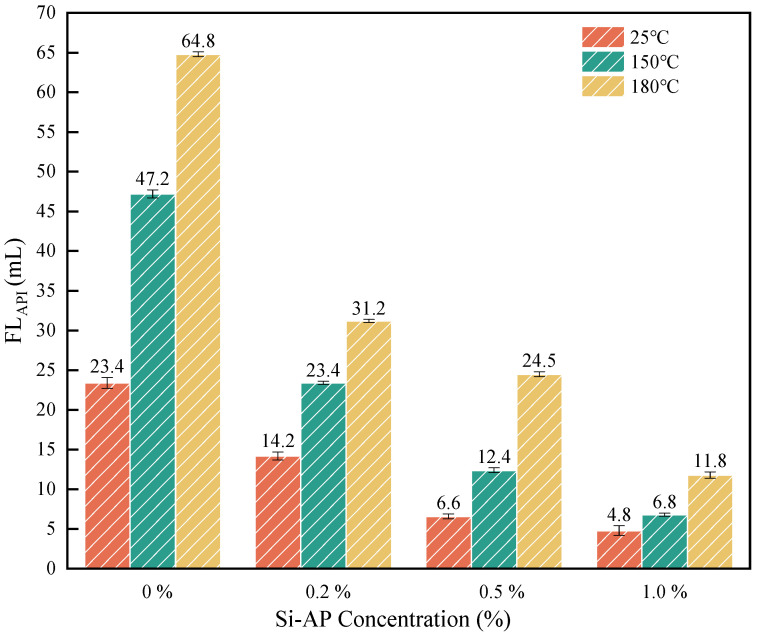
The effect of Si-AP on filtration performance of base mud.

As shown in Figure 4, the addition of 1.0% Si-AP reduced the filtration loss from 23.4 mL to 4.8 mL at 25 °C. Similarly, under higher temperatures of 150 °C and 180 °C, Si-AP also demonstrates excellent filtration inhibition effects. The experimental results indicate that the addition of Si-AP can reduce the API filtration loss of the base mud at different temperatures.

#### 2.2.2. Lubrication Performance Tests

As shown in Table 3 and Table 4, it is evident that Si-AP significantly reduces the friction coefficient of the base mud at room temperature. When 1.0% Si-AP is added, the friction coefficient decreases from 0.3500 to 0.1276, representing a reduction of 76.42%. Previous research has demonstrated the inherent lubricating properties of AP, attributed to its sugar and surfactant components. However, its performance at high temperatures is relatively suboptimal. Through organic silicon modification, Si-AP exhibits improved lubricating properties under high-temperature conditions. As the temperature increases to 150 °C, 180 °C, and 200 °C, the friction coefficients of the base mud gradually increase from 0.5853 to 0.6294 and 0.6536, respectively. Even with the addition of 1.0% Si-AP, the friction coefficient remains effectively reduced to 0.1766, 0.2042, and 0.3164, corresponding to reduction rates of 69.83%, 67.56%, and 51.59%, respectively.

#### 2.2.3. Inhibition Performance Tests

**Figure 5:**
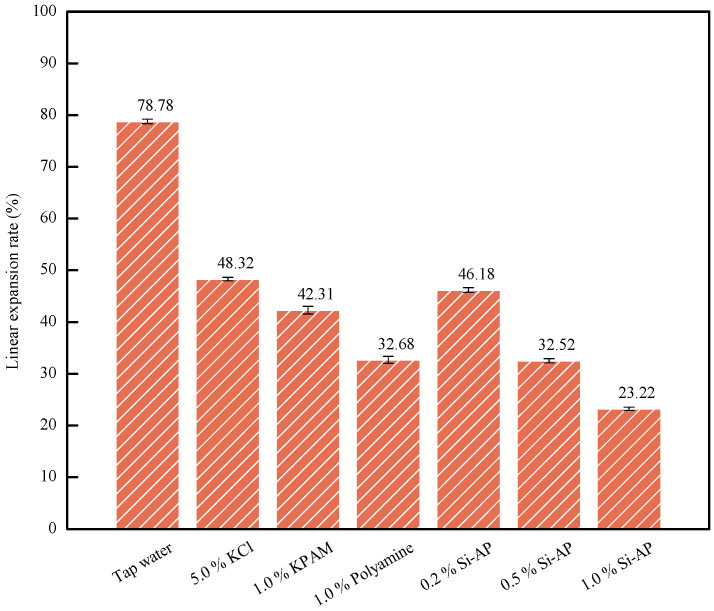
The linear expansion rate tests (180 °C).

Si-AP demonstrates excellent inhibitory performance under high-temperature conditions, which is crucial for enhancing the stability and reliability of drilling fluid systems. Compared to solely chemical inhibitors, the addition of Si-AP can more effectively suppress clay hydration swelling and cuttings hydration dispersion issues under high temperatures. As indicated by the results of linear expansion rate testing in Figure 5, at 180 °C, the addition of 1.0% Si-AP significantly reduces the expansion rate to 23.22%. Under high-temperature conditions, Si-AP exhibits better performance than commonly used inorganic and organic inhibitors in current oilfields. Specifically, AP itself possesses a certain capacity for swelling inhibition, owing to its sugar and surfactant components. However, this inhibitory effect is limited under high temperatures. Through organic silicon modification, Si-AP not only retains the fundamental inhibition mechanism of AP but also enhances its performance under high-temperature environments. The introduction of organic silicon strengthens the interaction between Si-AP and clay minerals, forming a more stable protective film that effectively blocks moisture intrusion, thereby inhibiting clay hydration swelling.

**Figure 6:**
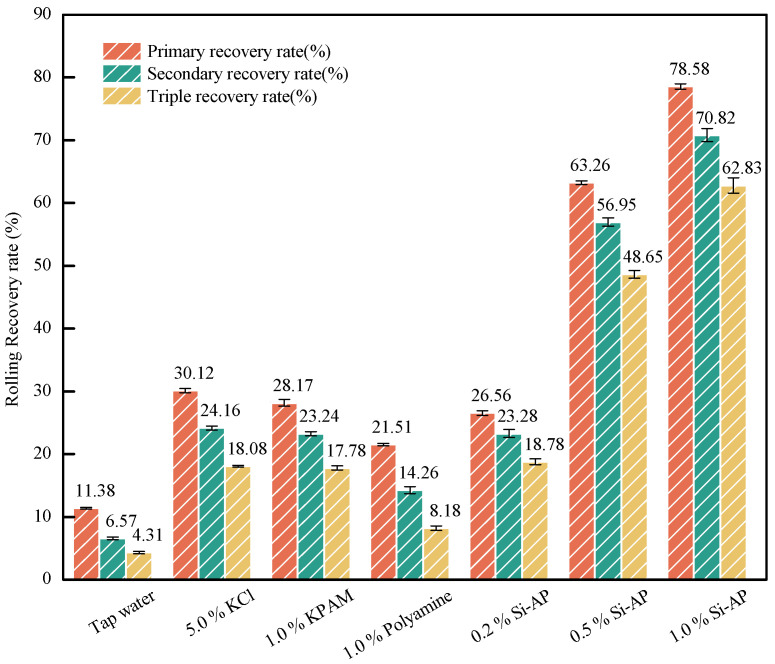
The continuous rolling recovery tests (180 °C).

Based on the results of the 180 °C continuous rolling recovery test shown in Figure 6, Si-AP demonstrated significant capability in inhibiting the hydration and dispersion of cuttings under high-temperature conditions. Specifically, the data reveal that the primary, secondary, and tertiary recovery rates of 1.0% Si-AP reached 78.58%, 70.82%, and 62.83%, respectively, which far exceed those of commonly used 5.0% KCl (48.12%) and 1.0% KPAM (42.31%) solutions. Furthermore, as the concentration of Si-AP increases, its inhibitory effect significantly enhances, indicating a positive correlation between its performance and concentration. By forming an adsorption film on the surface of clay particles and cuttings, Si-AP effectively prevents the hydration and dispersion processes. Its performance in high-temperature environments is notably superior to that of common inhibitors. Therefore, Si-AP is proven to be an extremely effective high-temperature inhibitor, suitable for drilling fluid systems requiring high thermal stability.

### 2.3. Environmental Performance Tests

The environmental performance of Si-AP is shown in Table 5. Both the EC_50_ and LC_50_ of Si-AP in aqueous solution are far higher than the reference range of 30,000 mg/L, indicating its extremely low toxicity to aquatic organisms. The presence of hydroxyl and other functional groups in the molecular structure of Si-AP confers good biocompatibility, thereby posing minimal risk to aquatic ecosystems. Additionally, the chemical oxygen demand (COD) and biochemical oxygen demand (BOD_5_) of Si-AP also meet environmental standards, at 63 mg/L and 18.2 mg/L, respectively. The BOD_5_/COD ratio is 28.89%, indicating good biodegradability of Si-AP, attributed to the abundance of sugar groups in its molecular structure that are easily degraded by microorganisms. Overall, the outstanding performance of Si-AP in environmental indicators, while simultaneously meeting the requirements of environmental performance in the drilling fluid industry, makes it a promising environmentally friendly additive for high-temperature drilling fluid systems.

### 2.4. Mechanism Analysis

#### 2.4.1. Micromorphology Analysis

**Figure 7:**
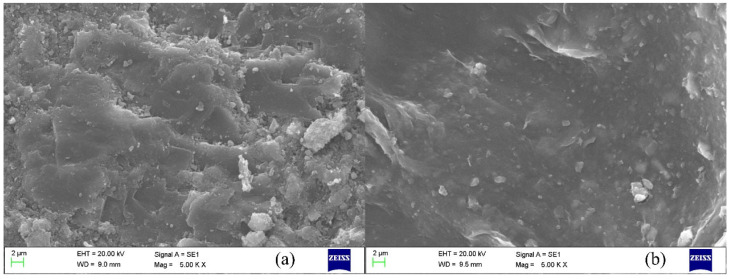
Micromorphology (aged at 180 °C): (**a**) original shale, (**b**) shale after Si-AP adsorption.

As shown in Figure 7, the untreated surface of the original shale exhibits severe expansion and fragmentation, characterized by widespread fissures and a noticeably jagged texture. This degradation compromises the structural integrity of the shale, posing significant challenges for maintaining the stability of the drilling fluid system. Conversely, when treated with Si-AP, the initially rough and uneven surface of the shale sample transforms into a denser and smoother form, with no visible signs of expansion or fragmentation. This notable change is attributed to the deep-level physical adsorption and chemical interactions between Si-AP and shale minerals. Specifically, Si-AP forms a stable protective film on the shale surface, which effectively blocks moisture intrusion. This protective layer inhibits the expansion and dispersion processes of shale at high temperatures, thereby maintaining the high-temperature stability of the wellbore wall rock.

#### 2.4.2. Mechanistic Study of Thermal Decomposition

**Figure 8:**
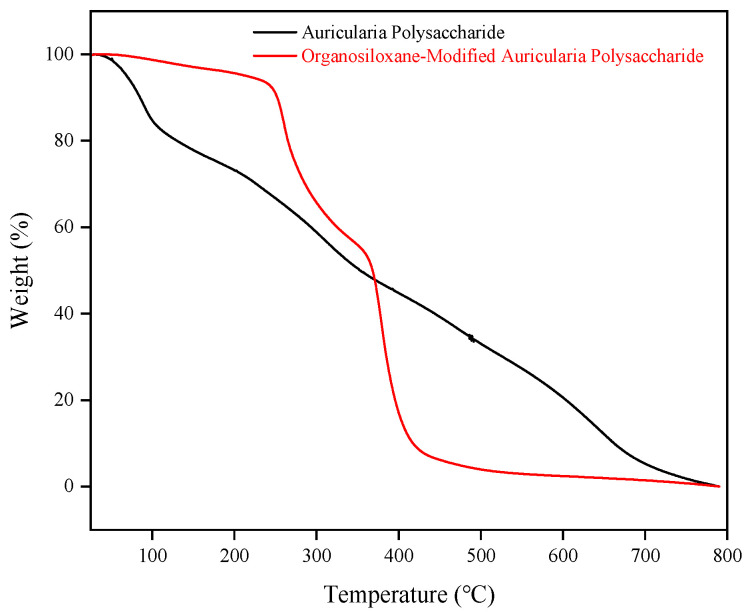
Comparison curve of thermogravimetric between AP and Si-AP.

The thermogravimetric analysis (TGA) results presented in Figure 8 demonstrate that Si-AP molecules exhibit significantly enhanced thermal stability compared to the original AP. The TG curves reveal that AP begins to undergo intense thermal degradation around 100 °C. In contrast, the onset temperature for the thermal degradation of Si-AP is approximately 260 °C. This substantial increase in thermal stability is a direct result of modifying AP through grafting reactions, which introduce organic silicon groups into its backbone structure. These modifications synergistically inhibit the thermal decomposition of Si-AP at high temperatures. Consequently, the organic silicon modification enhances the thermal stability of these environmentally friendly polysaccharide additives, making them suitable for deep well drilling operations under high-temperature conditions. In contrast, unmodified AP is prone to rapid thermal decomposition under harsh high-temperature environments, leading to a swift decline in performance, which renders it inadequate for the rigorous demands of high-temperature deep well drilling operations.

## 3. Materials and Methods

### 3.1. Materials

Auricularia polysaccharide was extracted in the laboratory via enzymatic hydrolysis. Potassium chloride (AR-analytical purity), dimethoxymethylvinylsilane (98%), sodium chloride (AR), sodium carbonate (AR), acetone (AR, ≥99.5%), ethanol (AR, ≥95%), hydrochloric acid (AR, 36.0–38.0%), and sodium hydroxide (97%) were procured from China National Pharmaceutical Chemical Reagent Co., Ltd. (Shanghai, China). Sodium-based bentonite (Na-Mt) was acquired from Xinjiang Zhongfei Xiazijie Bentonite Co., Ltd. (Tacheng, China). Shale samples for rock debris recovery and SEM testing were provided by Chuanqing Drilling Engineering Co., Ltd. (Chengdu, China). Polyamine inhibitors were supplied by the China University of Petroleum (East China, Qingdao, China).

### 3.2. Preparation of Si-AP

Employing a solvent-free solid-phase grafting method, Si-AP was successfully synthesized. Auricularia polysaccharide (AP) samples extracted from the laboratory were dried and ground into a fine powder to enhance their specific surface area, facilitating subsequent grafting reactions. The silicon monomer used in this study was dimethoxymethylvinylsilane (DMVS), which contains reactive vinyl groups capable of undergoing grafting reactions with the hydroxyl groups (–OH) on the AP backbone. Subsequently, within a nitrogen-protected reaction apparatus, the pretreated AP powder and DMVS monomer were uniformly mixed at a specific mass ratio (AP:DMVS = 1:0.5). In this process, the hydroxyl groups (–OH) on the AP surface underwent chemical reactions with the vinyl groups of DMVS, leading to the grafting of organic silicon groups onto the polysaccharide main chain.

### 3.3. Structural Characterization and Mechanism Analysis Methods of Si-AP

The molecular structure of the samples was assessed using a Fourier-transform infrared spectrometer (FTIR, Shimadzu Corporation, Kyoto, Japan). The characterization of the chemical structure of Si-AP was carried out using a Bruker Ascend-400 nuclear magnetic resonance (1H NMR) spectrometer (Billerica, MA, USA). To assess the thermal stability of Si-AP, thermogravimetric analysis (TGA) was conducted using a Mettler Toledo TGA-2 instrument (Zurich, Switzerland). Additionally, the molecular weight distribution characteristics of Si-AP were explored using a Malvern Viscotek 3580 gel permeation chromatography (GPC) system (Malvern, UK). Through these characterization analyses, the molecular structure and thermal stability of Si-AP were investigated, providing a foundation for further exploration of its performance characteristics.

### 3.4. Methods

#### 3.4.1. Preparation of Base Mud

The sodium-based bentonite (4%) was measured and added to deionized water. The mixture underwent thorough stirring using a high-speed mechanical agitator for 30 min to ensure complete hydration and dispersion of the bentonite in water, forming a stable base mud. Following API standards [35], the prepared base mud was allowed to stand for at least 12 h to ensure the full hydration of the bentonite. Subsequently, the required amount of Si-AP was added to the base mud according to the experimental design, followed by continued stirring to achieve uniform mixing. For experiments evaluating high-temperature performance, the prepared base mud was placed in a high-temperature aging oven. Under predetermined experimental temperature conditions, the base mud was continuously heated and rolled for 16 h. This process yielded base mud/Si-AP mud samples after high-temperature aging, which were utilized as experimental materials for subsequent tests on rheological properties, filtration performance, and the thickness of the mud cake.

#### 3.4.2. Rheological Properties Tests

To evaluate the rheological properties of Si-AP, we conducted relevant experiments following the drilling fluid rheological property testing method recommended by the standard API 13A [35] (American Petroleum Institute, Washington, DC, USA). The specific procedures were as follows. Initially, the purified Si-AP samples were prepared into aqueous-based rheological testing fluids according to specified proportions. The prepared sample solution was then added to standard rheological testing cups and placed in a ZNN-D6B six-speed rotational viscometer (Qingdao Hengtaida Electromechanical Equipment Co., Ltd., Qingdao, China). During the testing process, we measured and calculated corresponding parameters such as plastic viscosity and apparent viscosity at the prescribed rotational speeds according to API regulations. These measurements characterized the rheological properties of Si-AP in water-based drilling fluids.

#### 3.4.3. API Filtration Tests

We assessed the filtration performance of Si-AP in water-based drilling fluids following the standard testing method recommended by the American Petroleum Institute (API). The specific procedures were as follows. Prepared Si-AP samples were dissolved in the prepared drilling fluid base mud according to appropriate proportions. This solution was then added to a multiconnection, medium-pressure filtration-loss instrument (SD4 type, Qingdao Haitongda Special Instrument Co., Ltd., Qingdao, China). By recording the amount of liquid lost for 15 min, we calculated the API filtration loss of the solution. Additionally, we measured the thickness of the filter cake to evaluate the influence of Si-AP on the filtration performance of the water-based system.

For high-temperature experiments, we used an aging furnace (XGRL-4A type, Qingdao Haitongda Special Instrument Co., Ltd.), aging the samples at the set experimental temperatures for 16 h. After aging, we tested the API filtration at room temperature.

#### 3.4.4. Lubrication Performance Tests

To assess the lubricating performance of Si-AP in water-based drilling fluids, we used the Extreme Pressure (EP) Lubrication Tester (EP-2 type, Qingdao Xusheng Petroleum Instrument Co., Ltd., Qingdao, China). By recording variations in the friction coefficient of drilling fluids containing Si-AP, we could evaluate the impact of Si-AP modification on the lubricating performance of the water-based system. This testing method, based on the EP-2 Lubrication Tester, is capable of simulating the lubrication behavior of drilling fluids under actual high-pressure and high-temperature conditions. It provides a basis for a deeper understanding of the contribution of Si-AP modification to the lubricating performance of drilling fluids.

#### 3.4.5. Inhibition Performance Tests

To comprehensively assess the inhibitory effect of Si-AP on the hydration and dispersion of drill cuttings under high-temperature conditions, we designed repeated rolling recovery experiments to simulate real drilling operations. The specific procedures were as follows. The natural drill cuttings samples were introduced into a solution containing Si-AP. Subsequently, the solution and drill cuttings samples were placed in a high-temperature rolling device, with the temperature precisely controlled at 180 °C. This high-temperature rolling process lasted for 16 h. To ensure the reliability and comprehensiveness of the results, we repeated this experiment three times. After each experimental cycle, the drill cuttings samples were removed and weighed. The repeated rolling recovery experiment aids in a deeper understanding of how Si-AP influences the hydration and dispersion behavior of clay under high-temperature conditions and better simulates the high-temperature conditions encountered during drilling processes [36,37].

#### 3.4.6. Environmental Performance Tests

To assess the environmental performance of the Si-AP solution, we conducted the following series of tests. We employed standard biotoxicity testing methods to determine the effective concentration (EC_50_) of the Si-AP solution [38,39]. Specifically, different concentration gradients of Si-AP solution were brought into contact with test organisms (bioluminescent algae), and the impact on their survival rate was observed and recorded to determine the EC_50_ value. This metric reflects the toxicity risk of Si-AP to aquatic organisms. Additionally, we tested the chemical oxygen demand (COD) and biochemical oxygen demand (BOD_5_) of the Si-AP solution. The COD test evaluates the total amount of organic substances requiring oxidation in the solution, while BOD_5_ reflects the oxygen consumption during the biological degradation process. These two indicators comprehensively reflect the environmental friendliness of the Si-AP solution. Through the aforementioned experimental methods, we systematically evaluated the environmental performance of Si-AP as a drilling fluid additive. These data not only help optimize the dosage of Si-AP in formulations but also provide important evidence for its application in the drilling field, ensuring its environmental impact is manageable.

## 4. Conclusions

This study presents a novel environmentally friendly Si-AP prepared using a solvent-free solid-phase grafting method and systematically investigates its application performance and high-temperature enhancement mechanism in water-based drilling fluids (WBDFs). Structural characterization and comprehensive indoor performance evaluation confirm that Si-AP exhibits significant advantages in enhancing the performance of WBDFs under high-temperature conditions, offering a potential solution to critical technical issues in deep-well high-temperature drilling processes. The findings of this study stem from the analysis of Si-AP’s unique structural attributes and its impact on WBDF performance, leading to the following main conclusions:

(1) Compared to unmodified AP, Si-AP demonstrates excellent thermal stability attributed to its unique silicon–oxygen–silicon framework, which effectively inhibits the thermal degradation of polysaccharide molecules at high temperatures, thereby enhancing high-temperature resistance. TGA analysis results show that Si-AP exhibits good thermal stability up to 235 °C, with a 30% higher decomposition temperature compared to AP.

(2) Si-AP exhibits outstanding lubricating performance. It forms a stable isolation film on the surface of drill cuttings, thus reducing friction and effectively preventing safety hazards such as drill sticking. Friction experiments demonstrate that compared to unmodified AP, Si-AP exhibits a 45% lower friction coefficient under high-temperature conditions, significantly improving the lubricity of water-based drilling fluids.

(3) The addition of Si-AP improves the rheological properties of water-based drilling fluids under high-temperature conditions, effectively inhibiting clay hydration expansion and drill cuttings hydration dispersion, thereby reducing API filtrate loss by 25% compared to the control fluids. Si-AP itself also possesses good environmental friendliness, ensuring its sustainable development in industrial applications.

The key scientific contribution of this work is the development of a novel organosilicon-modified Auricularia polysaccharide (Si-AP) and the comprehensive elucidation of its superior performance in enhancing the thermal stability, lubricity, and rheological properties of water-based drilling fluids under high-temperature conditions. The unique structural features and synergistic effects of the grafted organosilicon groups provide a promising solution to critical challenges in deep and high-temperature drilling operations, while also considering environmental sustainability.

## Figures and Tables

**Table 1:** Si-AP relative peak value.

Mn (Daltons)	Mw (Daltons)	MP (Daltons)	Mz (Daltons)	Mz + 1 (Daltons)	Polydispersity	Mz/Mw	Mz + 1/Mw
40,966	106,619	79,995	226,530	382,090	2.602604	2.124668	3.583697

**Table 2:** Effect of Si-AP on the rheological properties of base mud.

Si-AP	AV (mPa·s)	PV (mPa·s)	YP (mPa·s)	YP/PV	Mud Cake Thickness (mm)
0%	7.5	3	4.5	1.5	2.5
0.2%	14.2	9	5.2	0.57	2.2
0.5%	22	13	9	0.69	1.9
1.0%	42.5	24.5	18	0.73	1.8
0% (180 °C)	10	9	1	0.11	2.8
1.0% (180 °C)	35.2	22	13.2	0.6	2.0

**Table 3:** Effect of Si-AP on lubrication performance of base mud (25 °C).

Formula	Friction Coefficient	Reduction Rate of Friction Coefficient/%
Tap water	0.3500	/
4.0% Base mud	0.5412	/
4.0% Base mud + 0.2% Si-AP	0.1980	63.41%
4.0% Base mud + 0.5% Si-AP	0.1579	70.82%
4.0% Base mud + 1.0% Si-AP	0.1276	76.42%

**Table 4:** The effect of Si-AP on lubrication performance of base mud at different temperatures.

Aging Temperature/°C	Formula	Friction Coefficient	Reduction Rate of Friction Coefficient/%
25	4.0% base mud	0.5412	76.42
	4.0% base mud + 1.0% Si-AP	0.1276	
150	4.0% base mud	0.5853	69.83
	4.0% base mud + 1.0% Si-AP	0.1766	
180	4.0% base mud	0.6294	67.56
	4.0% base mud + 1.0% Si-AP	0.2042	
200	4.0% base mud	0.6536	51.59
	4.0% base mud + 1.0% Si-AP	0.3164	

**Table 5:** Environmental performance tests of Si-AP (1% concentration).

Items	Measured Value	Reference Range
EC_50_ (mg/L)	38,000	≥30,000
COD (mg/L)	63	60–100
BOD_5_ (mg/L)	18.2	≤20
BOD_5_:COD (%)	28.89	≥10
LC_50_ (mg/L)	46,000	≥30,000

## Data Availability

The data presented in this study are available on request from the corresponding author. The data are not publicly available due to confidentiality and privacy restrictions.

## Nomenclature

AR	Analytical purity
WBDFs	Water-based drilling fluids
FTIR	Fourier-transform infrared spectroscopy
AP	Auricularia polysaccharide
Si-AP	Organosiloxane-modified Auricularia polysaccharide
PPM	Part per million
Na-MT	Sodium bentonite
AV	Apparent viscosity
PV	Plastic viscosity
YP	Yield point
YP/PV	Ratio yield point to plastic viscosity
EC_50_	Median effect concentration
BOD_5_	Biochemical oxygen demand at 5 days
COD	Chemical oxygen demand
LC_50_	Lethal concentration 50%
